# Peripheral–central immune interactions in Parkinson’s disease: insights into innate and adaptive immunity

**DOI:** 10.3389/fnmol.2025.1682006

**Published:** 2025-10-08

**Authors:** Jarika Jahan Tumpa, Qurat Ul Ain Hayder, Nowshin Sharmily Maisa, Md. Nazmul Islam

**Affiliations:** ^1^Department of Pharmaceutical Sciences, North South University, Dhaka, Bangladesh; ^2^Department of Molecular Genetics, Brain Research Institute (BRI), Niigata University, Niigata, Japan; ^3^Department of Bioscience, COMSATS University Islamabad (CUI), Islamabad, Pakistan; ^4^Department of Biochemistry and Microbiology, North South University, Dhaka, Bangladesh; ^5^Department of Neuroscience of Disease, Brain Research Institute, Niigata University, Niigata, Japan; ^6^Department of Microbiology, Noakhali Science and Technology University, Noakhali, Bangladesh

**Keywords:** Parkinson diseases, immune system, peripheral and central nervous system immune response, neuroinflammation, adaptive immunity

## Abstract

Parkinson’s disease (PD) is a complex, multisystem disorder characterized by chronic inflammatory processes. The development of effective immunomodulatory therapies for PD requires a novel and comprehensive understanding of the coordinated interactions between central and peripheral innate and adaptive immune responses that are initiated and evolve throughout disease onset and progression. Immune system dysregulation is a defining feature of PD, with a substantial body of evidence indicating pathological alterations in both central and peripheral immune responses that evolve dynamically over the course of the disease. In PD, central inflammation is defined by the activation of astrocytes, microglia and T-cell responses within the central nervous system. In contrast, peripheral inflammation involves the activation of T-cell signaling and innate immune cells in the enteric nervous system, circulatory system and gastrointestinal tract. However, the underlying mechanisms of this inflammation in PD-associated diseases remain unclear, and identifying the initial stages of these diseases is a major unmet need. This review seeks to address this question by highlighting and discussing the central and peripheral systems through the collection of current data from clinical evidence and findings from experiments.

## 1 Introduction

There is now a broad consensus that the maintenance of the brain and other organ systems is of paramount importance to brain health. This underscores the importance of bidirectional communication. The brain is considered a highly immune-specialized organ that contains dedicated immune cells. Within the classification of neurodegenerative disorders, PD ranks as the second most prevalent condition after Alzheimer’s disease (AD). Projections indicate that its prevalence will double over the ensuing generation. Similarly, both diseases present analogous pathological characteristics, including the accumulation of protein aggregates, such as *α*-synuclein, amyloid-*β*, and tau, within the central nervous system (CNS). This accumulation primarily results from the aging process (Tolosa et al., 2021).

In PD pathology, the presence of neuronal inclusions, including anomalous, aggregated or misfolded *α*-synuclein (α-syn) in neurons, known as Lewy bodies (LBs) and Lewy neurites, are observed, accompanied by cell loss in the substantia nigra and other regions of the brain. The mechanism underlying the spread of Lewy pathology is thought to originate in the caudal brainstem, with subsequent progression through the upper brainstem, limbic regions, and ultimately the neocortex (Braak et al., 2002). However, this progression may not occur uniformly in all cases. Recent studies strongly suggested that prion-like *α*-syn is transferred from cell to cell and permissive templating of α-syn, which is considered a key mechanism of PD progression (Steiner et al., 2018). A further crucial aspect is the degeneration of dopaminergic (DA) neurons located in the basal ganglia, which results in the gradual onset of motor symptoms, including resting tremors, slowness or absence of movement, and postural instability. Furthermore, Lewy bodies have been observed in the ganglia and axons of the peripheral and enteric nervous systems (Braak et al., 2006). This evidence suggests that PD is a disease of the CNS.

The consistent findings obtained with *in vivo* and *in vitro* models suggested that inflammation plays a pivotal role in the pathogenesis of PD. According to the results of postmortem brain samples from patients with PD, microglia, which are the resident tissue macrophages of the CNS, initiate innate immune responses upon activation by various stimuli. This activation is accompanied by the infiltration of T cells and peripheral monocytes into the brain parenchyma (Tufekci et al., 2012). To ensure proper tissue homeostasis and prevent collateral damage during inflammatory responses, inflammatory responses in the CNS must be resolved and terminated, similar to those observed in the peripheral immune system. However, microglia and T cells are found in close proximity to *α*-syn aggregates and regions of neurodegeneration. This observation supports the involvement of both innate and adaptive immune responses in the pathophysiology of PD.

## 2 Cross-talk between peripheral and central immune systems

The peripheral immune system consists of lymphatic cells, including T cells, and B cells (Yan et al., 2021) which remain inactive under resting conditions. However, during antigen introduction, the cells become activated and provide a specific immune response. While the peripheral immune system is considered reactive, the central immune system is proactive, meaning that it continuously regulates brain immunity. The central immune system is composed not only of neurons and glial cells but also of other immune cells (Zang et al., 2022). It acts by integrating external trauma or chronic inflammation into its routine, adapts and provides immune feedback. However, in chronic inflammation, this ongoing immune feedback loop can lead to neuronal damage. Crosstalk between these two immune systems is considered a pivotal factor in the progression of PD (Passaro et al., 2021). The idea of mutual influences develops from the interconnection of reactions seen in the brain and in the periphery when the counterpart is injured. For example, CNS disruption makes one prone to inflammation (Bloom et al., 2020). On the other hand, bacterial endotoxins that cause systemic inflammation can weaken the blood–brain barrier, allowing immune cells, cytokines, and toxins to enter the brain and create neuronal damage via different pathways (Millán Solano et al., 2023). Our focus in this review is how peripheral and central immune dysregulation might influence neuroinflammation in PD.

A key aspect of modern research on PD is the growing recognition that the peripheral immune system actively interacts with the central immune system, particularly microglia. This communication occurs mainly through cytokine signaling and cellular migration, which together establish and sustain a chronic inflammatory state.

## 3 Systemic immune signals and PD progression

PD is a progressive multisystemic neurodegenerative disorder characterized by the preferential dysfunction and death of dopaminergic neurons and the presence of Lewy bodies in both dopaminergic and nondopaminergic brain areas. This trait was previously thought to be a movement disorder characterized by motor deficit, resting tremor, and rigidity of the neck and limbs and has now been established as a multisystem disorder resulting in neuroinflammation and immune dysfunction that escalates non-motor symptoms such as dementia, sleep disorders, hyposmia, hallucination and gastrointestinal dysfunction (Tansey et al., 2022). The distinguishable feature of this disease is the formation of Lewy bodies, which are initiated by the misfolding of the *α*-synuclein protein, ultimately resulting in neural dysfunction. Evidence also suggests that neuroinflammation can occur during the onset and progression of PD (Coukos and Krainc, 2024). However, the insidious nature of PD often obstructs the identification of definitive onset. This challenge hinders the precise determination of the underlying causes of dopaminergic cell death. While genetic mutations in PARK genes, such as those encoding alpha-synuclein, PINK1, and LRRK2, account for a small percentage (5–10%) of PD cases, the majority are likely influenced by a complex interplay of age-related factors and environmental exposures (Pajares et al., 2020).

The CNS, once considered an immunologically privileged sanctuary, is now recognized as a complex microenvironment subject to immune modulation. The BBB, which is a crucial boundary, does not entirely shield the CNS from peripheral immune influences (Nau et al., 2010). Resident glial cells, including microglia and astrocytes, play important roles in maintaining CNS homeostasis. However, the release of pathogen-associated molecular patterns (PAMPs) and damage-associated molecular patterns (DAMPs) from protein aggregates can lead to microglial activation. This results in neurovascular unit alterations and persistent neuroinflammation. After triggering an inflammatory response, microglia can function either as the M1 phenotype (which has a proinflammatory effect) or the M2 phenotype (which has an immunosuppressive effect). The release of ATP, neuromelanin, m-calpain (by dying neurons), and CCL2 (by astrocytes) favors the M1 phenotype acquisition. DAMPs released by dying neurons induce genes encoding the NADPH oxidase system, reactive oxygen species and nitric oxide, hence causing a chronic inflammatory state. The M1 phenotype comprises a large, amoeboid cell body that overexpresses both proinflammatory mediators (IL1β, IL6, TNFα, and chemokines) and MHC molecules (Cebrian et al., 2014; Pajares et al., 2020).

**Figure 1:**
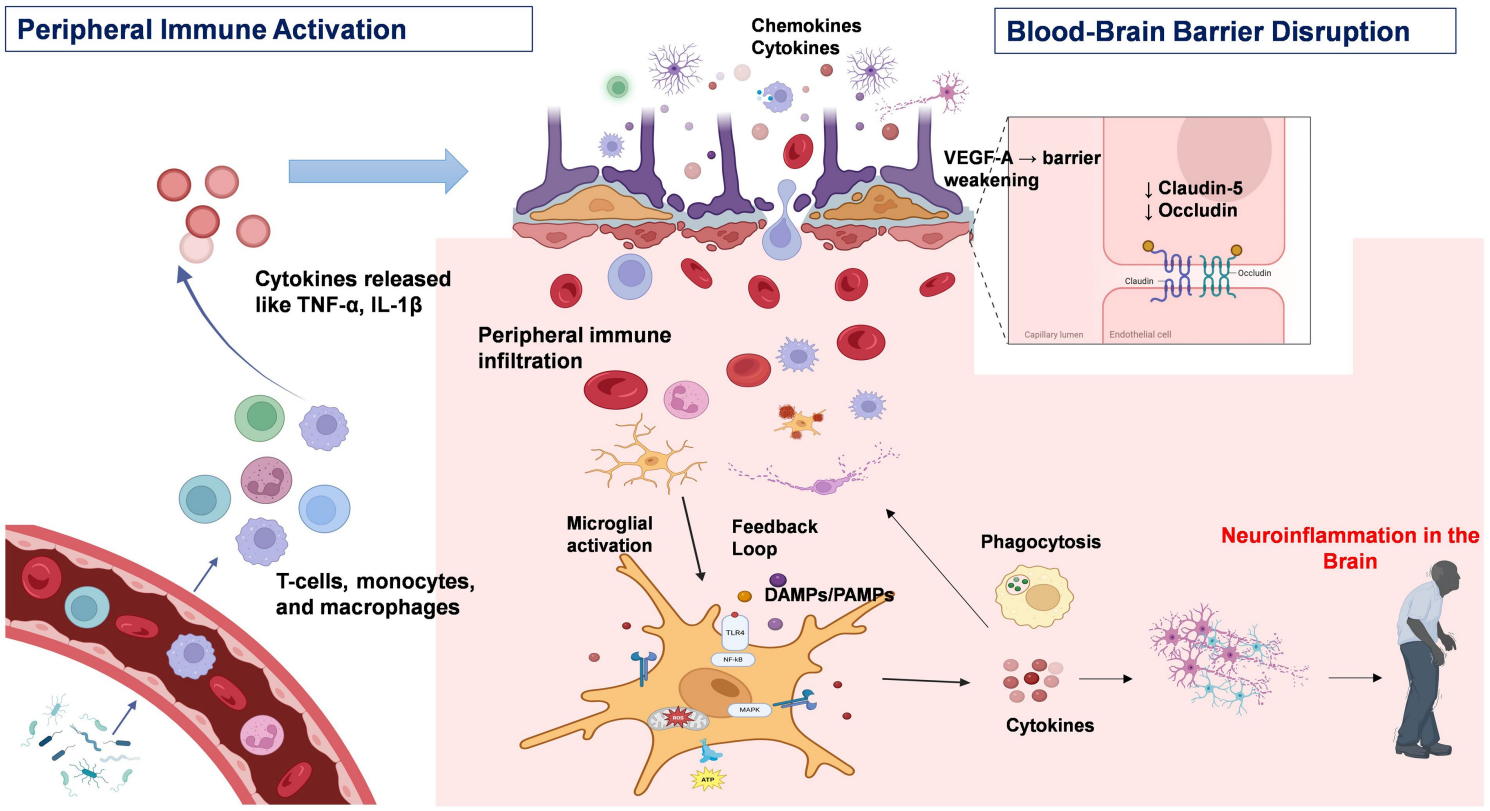
Peripheral immune activation and neuroinflammation in Parkinson’s disease. This figure depicts the immunopathological cascade underlying the progression from peripheral immune activation to central neuroinflammation in PD. Environmental triggers, such as bacterial endotoxins, stimulate peripheral immune cells—including T cells and macrophages—to produce pro-inflammatory cytokines such as TNF-*α*, IL-1β, and IL-6. Sustained cytokine release contributes to chronic systemic inflammation. These circulating cytokines can compromise the integrity of the blood–brain barrier (BBB) by downregulating tight junction proteins such as claudin-5 and occludin. Under inflammatory conditions, astrocytes further exacerbate BBB disruption through the release of vascular endothelial growth factor A (VEGF-A). Additionally, chemokines such as CCR2 promote leukocyte infiltration into the CNS via the compromised BBB. Within the CNS, infiltrating immune cells and circulating cytokines activate resident microglia. The activation of intracellular signaling pathways, including the NF-κB and MAPK pathways, within microglia amplifies the neuroinflammatory response, leading to increased production of pro-inflammatory mediators and reactive oxygen species (ROS). Chronic microglial activation enhances phagocytic activity and establishes a feedforward loop that perpetuates both peripheral and central immune responses, thereby accelerating neurodegeneration and disease progression in PD. Created with BioRender.com.

This phenotype is responsible for modifying the permeability of the BBB, which results in a reinforced local inflammatory response. The scarcity of anti-inflammatory mediators such as IL4 and IL10, pro-resolving mediators (lipoxins, resolvins, protectins and maresins) hinders the negative feedback mechanism that occurs under physiological conditions. MHC molecule makes the neurons vulnerable to cytotoxic T cells. Therefore, neurons incubated with M1 microglia result in cell death due to heightened expression of said molecules. However, when both the M1 and M2 phenotypes are present, neurotoxicity is reversed. Furthermore, the presence of a limited number of CD8^+^ T lymphocytes in close proximity to degenerating nigral neurons, in conjunction with the occurrence of components of the classical or antibody-triggered complement cascade within nigral Lewy bodies in patients diagnosed with PD, suggests the potential involvement of activation of adaptive immune responses in PD in the pathological process (Yamada et al., 1992). In the Postmortem sample analysis of PD brains has revealed the presence of immunoglobulin (IgG) binding to dopaminergic neurons in the SN and the presence of IgG-binding receptors (FcγRI) on microglia. Furthermore, an increase in peripheral CD3-positive, CD4 bright-positive, CD8 dull-positive lymphocytes has been observed in patients with PD (Hisanaga et al., 2001). The question of the antigens responsible for the activation of the microglial and adaptive immune response in PD remains unknown. However, on the Figure 1 shows the potential role of peripheral inflammation in compromising the integrity of the BBB, which may contribute to the onset or progression of CNS disorders such as PD. Disruption of the BBB allows potentially harmful substances and immune cells to penetrate the brain, thereby threatening neural homeostasis and function. A detailed understanding of the molecular and cellular mechanisms through which peripheral inflammation leads to BBB impairment is essential for developing therapeutic approaches aimed at preserving CNS diseases.

### 3.1 Cytokine signaling pathways

Activated peripheral immune cells, including T cells and macrophages, produce pro-inflammatory cytokines that play a central role in mediating systemic inflammation (Dzamko, 2023; Kany et al., 2019). This process is triggered when antigens derived from pathogens, damaged tissue, or bacterial endotoxins activate both innate and adaptive immune responses, resulting in prolonged inflammatory signaling (Wang et al., 2024a). Under normal conditions, peripheral immune activation serves as a protective response against pathogens and tissue damage. However, pro-inflammatory cytokines such as TNF-*α*, IL-1β, and IL-6 can disturb homeostasis during chronic inflammation, potentially contributing to neurodegeneration (Dzamko, 2023).

A central aspect of cytokine-mediated cross-talk is the disruption of the BBB. The BBB acts as a critical shield that protects the CNS from potentially harmful peripheral factors (Archie et al., 2021). However, in PD, the integrity of the BBB is compromised by multiple known mechanisms. Elevated levels of cytokines, including IL-1β, IL-6, IL-9, IL-17, IFN-*γ*, TNF-α, and CCL2, can alter the expression and localization of tight junction proteins, such as claudins, especially claudin-5, thereby compromising BBB integrity (Larochelle et al., 2011). Astrocytes, in response to inflammatory stimuli, can release either proinflammatory or anti-inflammatory mediators, which directly affect BBB function (Farfara et al., 2019). For example, under inflammatory conditions, they secrete vascular endothelial growth factor A, which downregulates occludin and claudin-5 expression, making the BBB permeable to peripheral lymphocytes (Argaw et al., 2012). Furthermore, the expression of chemokine receptors, such as CCR2, increases in response to inflammatory stimuli, which signals that immune cells migrate toward the CNS (Amason et al., 2024; David and Kubes, 2019).

Once within the CNS, proinflammatory cytokines activate resident microglia (Gao et al., 2023). These activated microglia release a variety of pro-inflammatory cytokines, such as TNF*α*, IL-6, IL-1β, IFN-*γ* and reactive oxygen species, perpetuating a cycle of inflammation and cellular damage that stimulates NF-κB cell death pathways (Flood et al., 2011; Gao et al., 2023). NF-κB is an essential transcription factor that controls the expression of several inflammatory genes, and when activated, it increases the production of cytokines and chemokines, helping to sustain the inflammatory response (Choi et al., 2018; Liu et al., 2017). Microglial activation also triggers the mitogen-activated protein kinase (MAPK) pathway (Zlokovic, 2008) which leads to altered gene expression and the release of additional inflammatory mediators (Choi et al., 2018). In PD patients, increased levels of cytokines such as TNF-α, IL-1β, IL-2, IL-6 and IFN-γ have been detected in both the serum and cerebrospinal fluid (CSF) (Fu et al., 2023; Hirano, 2021). Sustained microglial activation can drive a shift toward a neurotoxic M1 polarization, by increased expression of pro-inflammatory markers, further contributing to synaptic loss and neuronal damage (Piccioni et al., 2021). These intracellular cascades naturally lead to phagocytosis, which further promotes the release of pro-inflammatory cytokines that can attract more peripheral immune cells into the brain, creating a feedback loop that amplifies inflammation.

### 3.2 Cellular migration and pattern recognition

Immune cell movement alongside cytokine signaling is vital for communication between the peripheral immune system and the CNS. Peripheral immune cells express pattern recognition receptors (PRRs), such as Toll-like receptors (TLRs)(Chavan et al., 2017). TLRs assist them in recognizing PAMPs and DAMPs (Chavan et al., 2017; Li et al., 2021). Due to the activation of these receptors, a chain reaction of signals is initiated, strengthening the immune response and often leading to chronic inflammation in the nervous system. PRR activation leads to the formation of the NLRP3 inflammasome, which promotes the cleavage and activation of interleukin-1 beta (Palumbo et al., 2023), which plays a significant role in sustaining neuroinflammation in PD. Furthermore, in response to PAMPs and DAMPs, peripheral immune cells undergo functional modifications to increase their ability to infiltrate the CNS (Serna-Rodriguez et al., 2022). Taken together, these processes lead to a continuous influx of activated immune cells into the CNS, where they interact with resident glial cells and create a positive feedback loop.

A study revealed that LPS injection in wild-type mice increased immune sensitivity, increasing monocyte migration into the CNS. Mice with mutated *α*-synuclein developed Parkinson’s-like pathology, suggesting that peripheral α-syn release triggers immune activation and exacerbates neuroinflammation (Peralta Ramos et al., 2019). These mechanisms provide a clear framework for potential therapeutic targets that disrupt these harmful feedback loops. Managing inflammation should be a priority in PD treatment, as chronic peripheral immune activation contributes to BBB breakdown, neuroinflammation, and neuronal damage. Targeting peripheral immune dysregulation through anti-inflammatory cytokines and monoclonal antibodies against TNF-α or IL-1β could help reduce immune infiltration into the CNS, potentially slowing disease progression.

## 4 Role of gut-associated lymphoid tissue (GALT) in PD

GALT, or gut-associated lymphoid tissue, is primarily composed of isolated lymphoid follicles, Peyer’s patches (PPs) of the small intestine, and multi-follicular lymphoid nodes (Morbe et al., 2021). A GALT-mediated regulatory system plays a crucial role in maintaining the composition of the gut microbiome, modulating the gut-brain axis, and adapting to changes in the surrounding environment (Abo-Shaban et al., 2023). The GALT is considered the key antigen sampling site within the intestinal wall (Morbe et al., 2021).

### 4.1 Antigen sampling and immune surveillance in GALT

Antigen sampling in the intestine is the process by which the immune system detects and takes up antigens from the gut. Specialized cells such as M cells, goblet cells, and dendritic cells help transport or trap these antigens to initiate proper immune responses or maintain tolerance to harmless substances (Schulz and Pabst, 2013). GALT plays a crucial role in monitoring the intestinal environment by distinguishing between harmless gut microbes and potential pathogens (Bemark et al., 2024). Under normal conditions, immune surveillance assists in controlling excessive inflammatory responses. In PD, alterations in the gut microbiome, known as dysbiosis, disrupt this delicate balance (Munoz-Pinto et al., 2024). Multiple studies have reported that the bacterial DNA profile varies between patients with PD and normal patients (Cryan et al., 2020). These results suggested a higher level of pro-inflammatory microbial presence in the PD patient fecal matter rather than the anti-inflammatory microbiota (Keshavarzian et al., 2015).

A meta-analysis by Shen and colleagues revealed a reduction in the abundance of anti-inflammatory bacteria, such as *Lachnospiraceae,* which produces short-chain fatty acids (SCFAs), and a greater number of bacteria, such as *Ruminococcacea* and *Bifidobacteriaceae* (Shen et al., 2021). Dysbiosis can impair the integrity of the gut barrier, which is commonly referred to as the “leaky gut”(Termite et al., 2025). The leaky gut allows the translocation of bacterial products and antigens, leading to their interaction with the GALT (Tanwar et al., 2024). Due to this increased antigen exposure, GALT activates the production of pro-inflammatory cytokines such as IL-17 (Moretti et al., 2023; Saksida et al., 2018). These cytokines have systemic effects that ultimately contribute to neuroinflammation in the CNS, potentially contributing to the progression of neurodegenerative diseases, such as Parkinson’s disease.

### 4.2 Immune pathways involved in GALT-mediated inflammation

**Figure 2:**
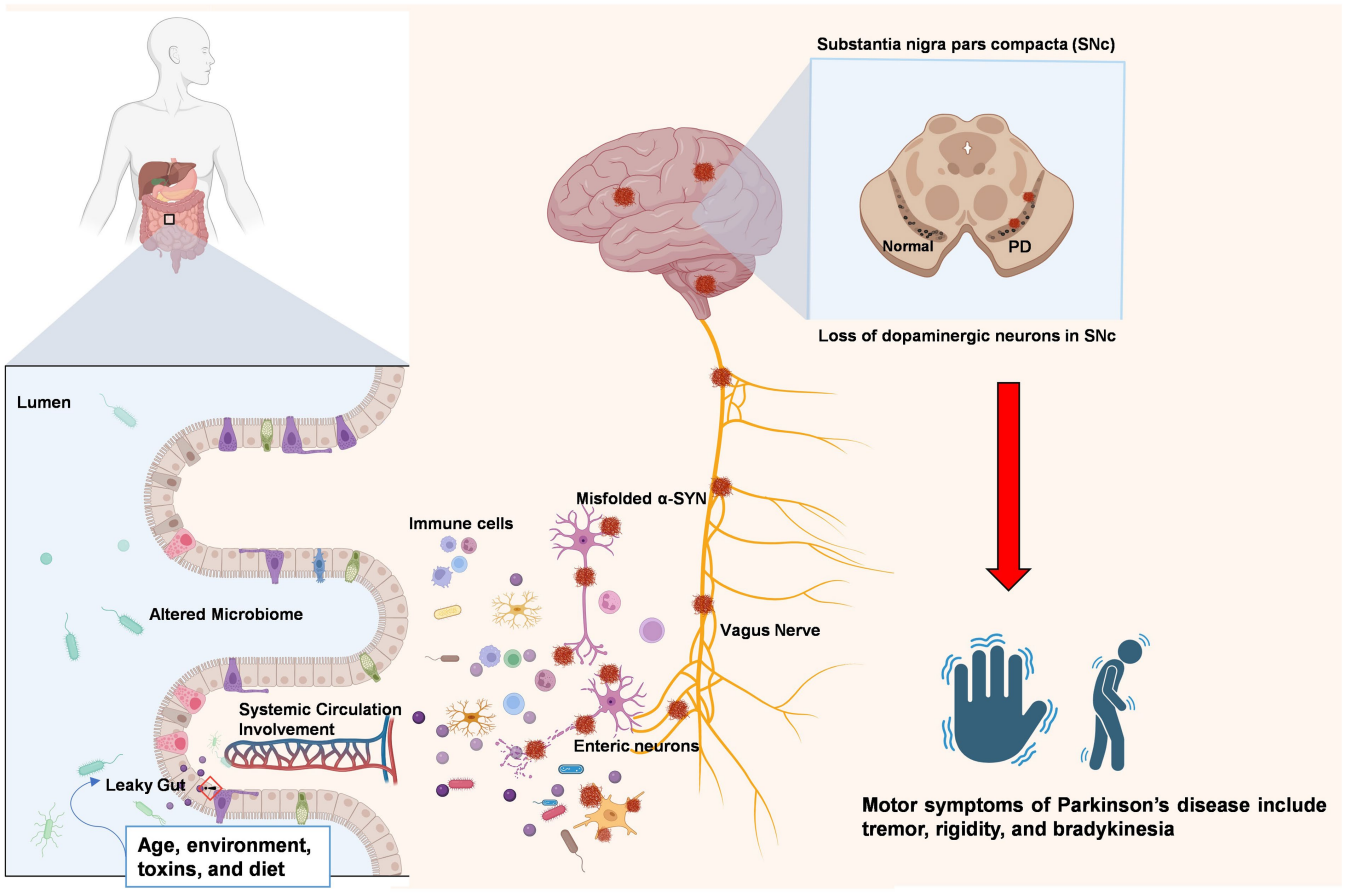
The gut–brain axis in Parkinson’s disease: from peripheral disruption to central neurodegeneration. The figure illustrates a proposed pathway for the transmission of misfolded α-synuclein (α-SYN) aggregates from the gastrointestinal system to the central nervous system via the vagus nerve. Environmental factors, including aging, diet, and exposure to toxins, have been demonstrated to alter the composition of the gut microbiota and compromise the intestinal barrier, a condition referred to as a “leaky gut.” This process enables the translocation of bacterial toxins and other inflammatory agents into the systemic circulation, thereby triggering immune responses and systemic inflammation. Local inflammation in the gut can affect the enteric nervous system (ENS), leading to the misfolding and accumulation of α-SYN aggregates. These aggregates may then propagate retrogradely along the vagus nerve to reach the substantia nigra pars compacta (SNc) in the brain, contributing to dopaminergic neuron degeneration and the onset of motor symptoms characteristic of PD. Created with BioRender.com.

In addition to maintaining gut homeostasis, the gut microbiota is also responsible for stimulating toll-like receptor (TLR) ligands (Caputi and Giron, 2018). These TLRs exert proinflammatory effects under certain conditions. Dysbiosis of the gut microbiota and damage to the gut epithelial barrier may instigate TLRs to initiate downstream signaling pathways that promote inflammation and oxidative stress in both the gut and brain of PD patients (Caputi and Giron, 2018). TLR2 works with TLR1 to recognize misfolded *α*-synuclein, which is released by neuronal cells. This recognition triggers downstream pathways involving proteins such as MyD88 and NF-κB that lead to the production of inflammatory cytokines such as TNF and IL-1β (Daniele et al., 2015). Studies have shown that the genetic absence of TLR4 signaling in mouse models protects the animal from neurodegeneration, highlighting its role in PD (Noelker et al., 2013). Similar results have also been reported for TLRs that modulate the complex NLRP3 inflammasome. A mouse model lacking the NLRP3 inflammasome showed less activation of inflammation-related proteins (Yan et al., 2015). However, Figure 2 illustrates the concept of the microbiota-gut-brain axis, which describes the bidirectional communication between the central nervous system and the gastrointestinal tract. In addition to the brain, key components of this axis include the gut microbiota, the intestinal epithelial barrier, the enteric nervous system, the gut-associated immune system, and the various pathways that link the gut and brain, such as neural circuits and humoral factors. The close interplay between the gut microbiota, GALT activation, and neuroinflammation in PD suggests multiple focal points for PD therapeutic targets. By modulating the gut microbiota and directly targeting GALT responses, it may be possible to develop early interventions that slow or prevent the progression of PD.

## 5 Metabolic and neural integration

As mentioned previously, scientists have reported a reduction in the number of anti-inflammatory bacteria that produce SCFAs in PD patients (Shen et al., 2021), including acetate (C2), propionate (C3) and butyrate (C4) organic acids, whose carbon chains are composed of fewer than six carbons(Fusco et al., 2023). These SCFAs help maintain intestinal integrity and permeability while promoting anti-inflammatory effects (Fusco et al., 2023; Silva et al., 2020). SCFAs also regulate the inhibition of histone deacetylase (HDAC), which affects the expression of genes related to neuroprotection (Mirzaei et al., 2021). SCFAs also impact immunity by directly affecting neutrophils, leading to their reduced production of reactive oxygen species (ROS), which promote their apoptosis (Fusco et al., 2023). They also reduce the chemotaxis of inflammatory cells toward the CNS by altering the expression of chemokine signals (Cox et al., 2009; Fusco et al., 2023). In PD, reduced SCFA production compromises these protective functions.

*In vivo* studies have shown that the gut microbiota stimulates the vagus nerve (Yu and Hsiao, 2021). Pro-inflammatory signals originating from GALT can be transmitted via this vagus nerve to central autonomic centers (Kasarello et al., 2023). Furthermore, aggregated alpha-synuclein might enter the brain through the direct pathway of the vagus nerve (Higinbotham and Kilbane, 2023).

## 6 Epigenetic regulation of immune responses in PD

**Figure 3:**
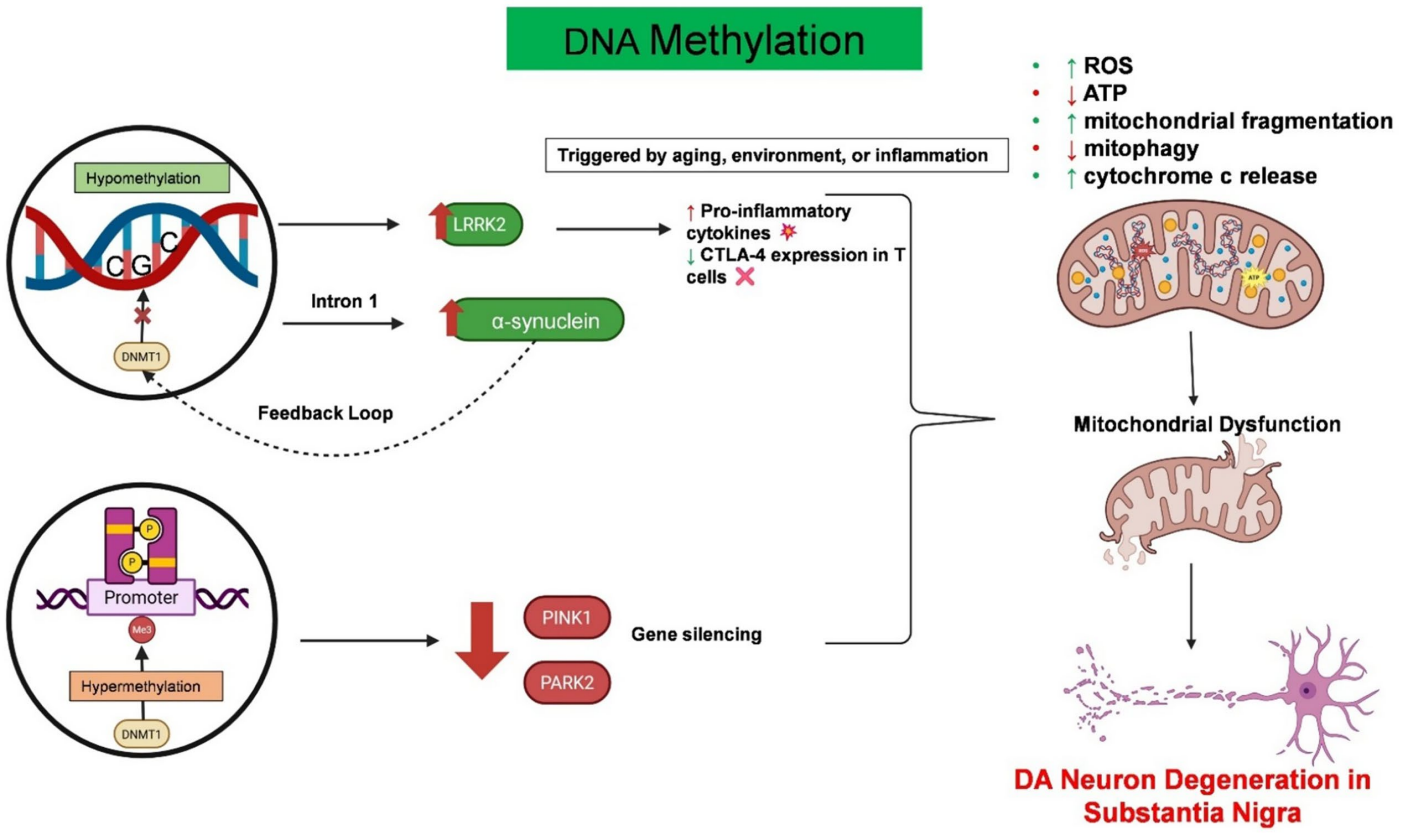
Epigenetic dysregulation of DNA methylation drives neuroinflammation and mitochondrial dysfunction in PD. This schematic summarizes the pathological consequences of aberrant DNA methylation in PD. Hypomethylation at intron 1 of the SNCA gene promotes the overexpression of α-synuclein. This further disrupts the function of DNMT1, creating a feedback loop. Similarly, hypomethylation of the LRRK2 gene in immune cells increases its expression, resulting in excessive cytokine release and reduced CTLA-4 expression in T cells. On the other hand, hypermethylation of the key mitophagy-regulating genes PARK2 and PINK1 leads to gene silencing, which further exacerbates mitochondrial dysfunction. Both conditions contribute to mitochondrial fragmentation, increased reactive oxygen species (ROS) production, mitophagy impairment, and cytochrome c release, which are the hallmarks of mitochondrial dysfunction. Created with BioRender.com.

**Figure 4:**
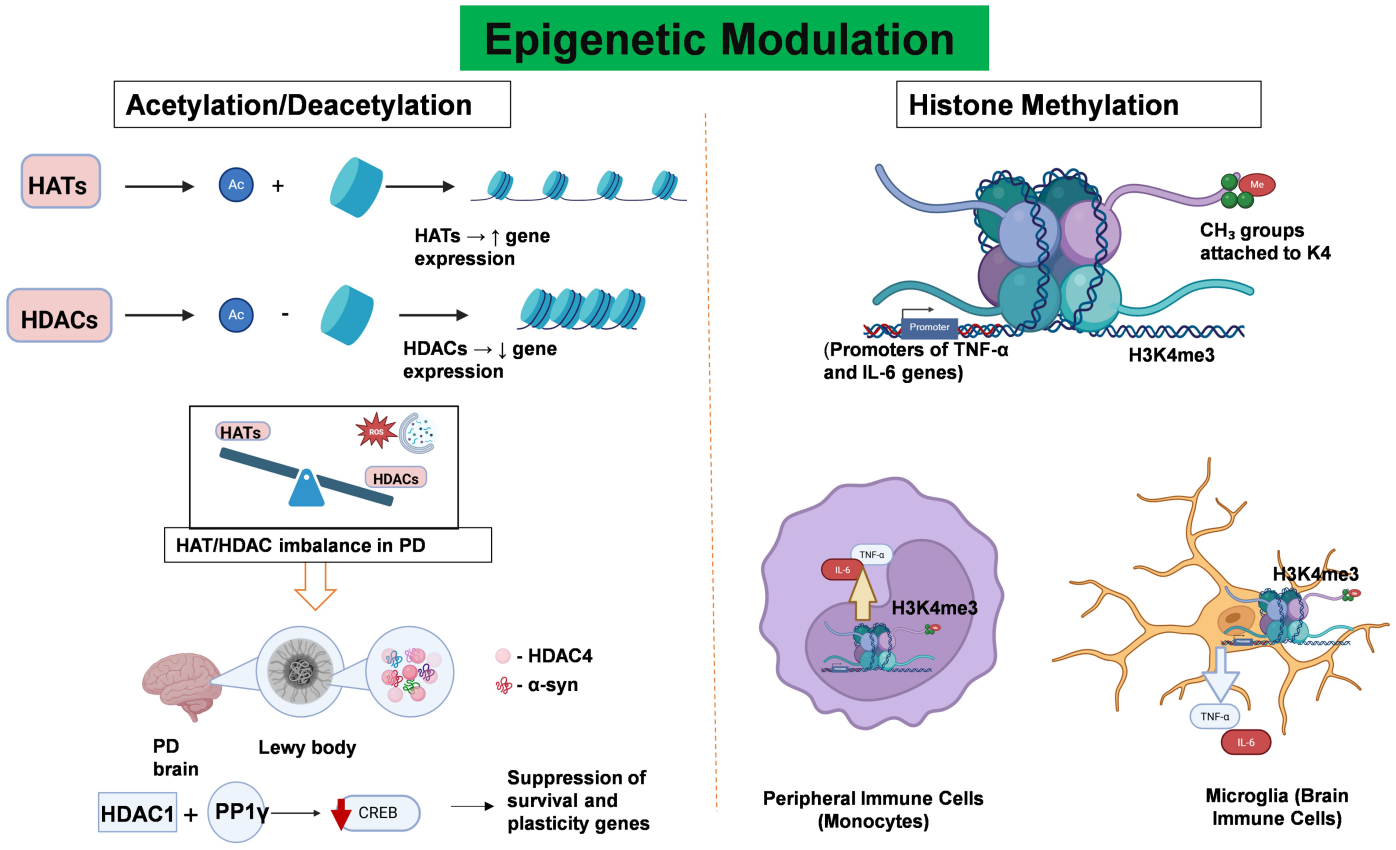
Epigenetic Modulation Regulates neuroinflammation and Gene Expression in PD. This figure illustrates the role of key histone modifications—acetylation/deacetylation and methylation—in regulating gene expression and neuroinflammatory responses in PD. Histone acetylation, which is mediated by histone acetyltransferases (HATs), facilitates transcriptional activation by relaxing chromatin structure through the addition of acetyl groups to histone tails. In contrast, histone deacetylases (HDACs) remove these acetyl groups, resulting in chromatin condensation and transcriptional repression. In PD, an imbalance between HAT and HDAC activity disrupts the transcription of genes critical for inflammation regulation and neuronal survival. Notably, HDAC4 colocalizes with α-synuclein aggregates in Lewy bodies, whereas HDAC1 interacts with the phosphatase PP1γ to suppress CREB activity, thereby contributing to neuronal dysfunction and degeneration. Abnormal histone methylation patterns, particularly trimethylation of histone H3 at lysine 4 (H3K4me3), have been implicated in the pathogenesis of PD. Elevated H3K4me3 levels in microglia and peripheral immune cells are associated with the upregulation of pro-inflammatory cytokines such as TNF-α, IL-1β, and IL-6, thereby promoting both systemic and central neuroinflammation. Created with BioRender.com.

Epigenetic regulation involves heritable changes in gene expression and chromatin architecture that occur without any alterations to the DNA nucleotide sequence itself (Wang et al., 2024b). Epigenetic modifications play essential roles in connecting environmental factors to gene expression and have been recognized as important regulators of immune responses in both the peripheral and central nervous systems in PD patients. These modifications, including DNA/RNA methylation, histone modification, chromatin remodeling, and non-coding RNAs (ncRNAs), regulate neuroinflammation in both PD forms (Rasheed et al., 2021). It is noteworthy that these epigenetic mechanisms act synergistically and do not independently regulate neuroinflammation in PD patients (Rasheed et al., 2021). Although the complete epigenetic landscape of PD has not yet been fully characterized, various epigenetic mechanisms such as DNA methylation, histone modification, and non-coding RNA regulation play critical roles in the molecular pathology of this disease. Figures 3, 4 illustrates the current understanding of DNA methylation and histone modification, two crucial epigenetic mechanisms involved in regulating gene expression and neurodegeneration within the peripheral immune system. In PD, epigenetic changes contribute to the onset and progression of neurodegeneration and neuronal death by modulating gene expression profiles (Table 1).

**Table 1:** Evidence of therapeutic targets, immune signaling mechanisms, and their limitations.

Immune mechanism/pathway	Therapeutic target(s)	Agents/clinical trials (phase, ID)	Limitations/challenges	References
α-Synuclein aggregation	Aggregated/misfolded α-synuclein	Vaccines (e.g., AFFITOPE PD01A/PD03A, UB-312; Phase I); mAbs (prasinezumab, cinpanemab, MEDI1341, Lu AF82422; Phases I–II)	No trials have shown clear efficacy yet; requires BBB penetration and patient selection; immune responses variable	Alfaidi et al. (2024)
Microglial/NLRP3 inflammasome	NLRP3 inflammasome, caspase-1, IL-1β	MCC950 (NLRP3 inhibitor, preclinical); ZYIL1 (NLRP3 inhibitor, Phase I)	Past drug (MCC950) halted by toxicity; human PD trials lacking; safety and selectivity concerns	Su et al. (2022)
LRRK2 kinase (microglia/monocytes)	LRRK2 kinase	DNL151/BiIB122 (Phase I/II completed); DNL201 (Phase I)	Only relevant for LRRK2-mutation carriers; long-term safety (lung, kidney) unknown; efficacy data pending	Jennings et al. (2022) and Jennings et al. (2023)
Adaptive immunity (T cells)	T cell activation	Sargramostim, Phase I	Non-specific effects; balancing neuroprotection vs. infection risk; limited clinical data	Gendelman et al. (2017)

### 6.1 DNA methylation

DNA methylation at the C5 position of cytosine within CpG dinucleotides is one of the primary epigenetic mechanisms (Kiselev et al., 2021). In PD, dysregulation of DNA methylation has been linked to both the overexpression of pathogenic proteins and immune dysregulation. For example, a decrease in DNA methylation in the brains of individuals with PD may be the result of the interaction between *α*-syn and DNA methyltransferase 1 (DNMT1) (Desplats et al., 2011). Furthermore, in sporadic PD patients, decreased methylation at SNCA intron 1 has been linked to dysregulated α-synuclein expression (Masliah et al., 2013). Additionally, DNA hypomethylation leads to LRRK2 overexpression in immune cells, which increases inflammation by increasing cytokine release and reducing CTLA-4 in T cells (Cook et al., 2017). Like α-synuclein, LRRK2 is a key genetic risk factor for PD that is localized at the mitochondrial membrane (Chen et al., 2022). Therefore, any mutation of this gene leads to mitochondrial damage and impaired degradation (Mortiboys et al., 2015). Hypermethylation of the PARK2 and PINK1 genes can impair mitochondrial autophagy and the accumulation of dysfunctional mitochondria, which results in the release of excessive reactive oxygen species (Barodia et al., 2017) (Figure 3).

In addition to the central nervous system, epigenetic regulation affects peripheral immune responses. Tumor necrosis factor alpha (TNF-*α*) is a principal pro-inflammatory cytokine that is upregulated in PD. Specific methylation of CpG dinucleotides in the TNF-α promoter decreases the binding of certain transcription factors, such as AP-2 and Sp1, which in turn reduces TNF-α promoter activity (Rasheed et al., 2021). Paradoxically, this reduced promoter activity may increase the sensitivity of dopaminergic neurons to TNF-α-mediated inflammation (Pieper et al., 2008). Similarly, hypomethylation of the IL-1β promoter induces microglial M1 activation, which amplifies neuroinflammation and neuronal damage (Lin et al., 2019; Wang et al., 2023a). Hypomethylation is also responsible for the increase in the binding of transcription factors, such as NF-κB, which increases the transcription of cytokine genes (Lu et al., 2024). Therefore, the hypomethylation-driven overexpression of cytokines such as TNF-*α* contributes to a chronic inflammatory environment within the nervous system.

### 6.2 Histone modifications

Histones are proteins that provide structure and organize DNA. Therefore, any modification to this protein might result in the up- or downregulation of genes. Acetylation, deacetylation, and methylation are particularly important in the context of neuroinflammation and neurodegenerative diseases such as PD.

### 6.3 Acetylation and deacetylation dynamics

Histone acetyltransferases (HATs) are enzymes that loosen the tightly packed DNA structure by adding acetyl groups to histone proteins (Gujral et al., 2020; Lee et al., 2020). This relaxed state makes it easier for genes to be transcribed, which promotes gene expression (Gujral et al., 2020). On the other hand, histone deacetylases (HDACs) work by removing acetyl groups from histones, which results in their positive charge. This tightens the chromatin, making it harder for transcription factors to access the DNA, leading to decreased gene expression (Milazzo et al., 2020).

In PD, an imbalance between HATs and HDACs is observed, which affects pathways that are linked to the immune system and inflammation (Harrison et al., 2018). Researchers have found that HDAC4 is highly expressed in affected brain regions and colocalizes with α-synuclein in Lewy bodies supporting their function in neurodegeneration (Wang et al., 2023b). Another study revealed that HDAC1 interacts with protein phosphatase 1γ (PP1γ), which shuts down the cyclic AMP response element binding (CREB) transcription factor, leading to neuron damage (Rasheed et al., 2021; Xu et al., 2022). The imbalance between HATs and HDACs, along with increased oxidative stress and impaired autophagy, further promotes neurodegeneration in PD.

### 6.4 Histone methylation

In contrast to acetylation, histone methylation can either activate or repress gene transcription, depending on the residue and context. Dysregulated histone methylation patterns have been observed in both microglia and peripheral immune cells in PD, indicating their role in disease progression. H3 methylation at the lysine position affects the SNCA gene, ultimately leading to altered expression of *α*-syn (Song et al., 2023). Additionally, increased H3K4 methylation in peripheral immune cells has been linked to increased transcription of pro-inflammatory cytokines such as TNF-α, IL-1β, and IL-6 (Wu et al., 2023). This promotes systemic inflammation, increasing BBB permeability and enabling inflammatory mediators to infiltrate the CNS, exacerbating neuroinflammation in the substantia nigra.

Epigenetic targeting of histone-modifying enzymes is a promising approach for PD treatment. HDAC inhibitors such as valproic acid may help restore gene expression balance and reduce inflammation (Watson et al., 2024). Histone methyltransferase and demethylase inhibitors can correct aberrant methylation patterns, potentially protecting dopaminergic neurons from degeneration. Understanding their interplay is essential for identifying precise therapeutic targets and designing more effective interventions.

## 7 Role of peripheral sensory neurons in immune activation in PD

Peripheral sensory neurons are a group of neurons that detect stimuli from the body’s periphery regions and deliver them to the brain via the peripheral nervous system. Currently, their role in immune regulation is gaining significant attention (Saraiva-Santos et al., 2024). These neurons interact with peripheral immune cells by releasing neuropeptides, engaging in receptor-mediated signaling, and altering ion channels (Yeo et al., 2022).

### 7.1 Neuropeptide release and receptor-mediated signaling

Sensory neurons release neuropeptides that act as messengers between the nervous and immune systems (Chavan et al., 2018). These messengers help manage inflammation and influence the environment in both the brain and peripheral tissues (Chu et al., 2020). For example, chronic pain and inflammation often prompt these neurons to release neurotransmitters such as substance P (SP) and calcitonin gene-related peptide (CGRP) (Ji et al., 2018). This release might lead to central sensitization and further neuroinflammation, potentially worsening PD.

SP works mainly by binding to the neurokinin-1 receptor (NK1R) on immune cells, such as macrophages, microglia, dendritic cells, and T cells (Singh et al., 2022; Zhu and Bhatia, 2023). Once NK1R is activated, immune cells start releasing cytokines such as TNF-*α*, IL-1β, and IL-6, which increases inflammation (Suvas, 2017). Similarly, CGRP signals through the CLR/RAMP1 receptor complex (Hay, 2019), increasing dendritic cell maturation and T-cell differentiation (Kim and Granstein, 2021; Yang et al., 2024). They also alter macrophage and neutrophil activity through changes in gene expression, leading to cytokine release and chemotaxis (Yang et al., 2024). These neuropeptide-immune interactions contribute to a pro-inflammatory environment that may worsen neurodegeneration in PD.

The MAPK/ERK pathway plays a key role in neurodegeneration by driving glial activation and inflammatory mediator production (Albert-Gasco et al., 2020). Neuropeptide activation triggers microglia and astrocytes to enter a pro-inflammatory state. This results in the release of increased levels of nitric oxide, cytokines (such as TNF-*α* and IL-1β), and chemokines (Albert-Gasco et al., 2020). These changes result from MAPK/ERK-dependent transcriptional reprogramming that amplifies both central and peripheral inflammatory responses (Bragelmann et al., 2021).

### 7.2 Ion channel modulation and neuronal excitability

Ion channels play a vital role in sensory neuron excitability and are responsible for the release of neuropeptides and immune cell activity (Spiers and Steinert, 2021; Starobova et al., 2024). Dysregulation of these channels can therefore directly impact inflammatory processes. The transient receptor potential vanilloid 1 (TRPV1) channel is an ion channel in sensory neurons that responds to heat and pain (Zhang et al., 2023). TRPV1 activates sensory neurons through calcium signaling and triggers the release of inflammatory neuropeptides (SP/CGRP) and cytokines (IL-6/IL-8) (Qu et al., 2023). This process activates glial cells and promotes neuroinflammation (Qu et al., 2023). In PD models, researchers have reported that TRPV1 is upregulated, increasing the release of SP and CGRP (Moore et al., 2023; Rahman et al., 2024). Moreover, TRPV1 affects immune system function by regulating calcium inside cells, which in turn influences cytokine production in immune cells (Chen et al., 2024). Therefore, this abnormal release of neuropeptides triggers the activation of microglia and attracts immune cells from the periphery, sustaining chronic neuroinflammation (Rahman et al., 2024).

Experimental studies have revealed that sensory neuron pathways can be novel therapeutic targets to reduce neuroinflammation in PD. Recent studies have shown that an NK1R antagonist (LY303870) reduces L-DOPA-induced dyskinesia (LID) in animal models (Yang et al., 2015). It blocks SP/NK1R signaling without compromising the motor benefits of L-DOPA (Yang et al., 2015). This finding supports the use of NK1R antagonists as potential adjunct therapies to manage LID in PD patients.

### 7.3 Role of *α*-synuclein on T cell

Within the adaptive immune system, T cells play a central role in pathogen clearance. During infection, they become activated, undergo clonal expansion, and play essential anti-infective functions role. Under normal conditions, T cell infiltration into the CNS is limited due to the immune privilege conferred by the BBB. However, over the past decades, accumulating evidence has demonstrated a significant association between T cell activation and the pathogenesis of neurodegenerative diseases, particularly AD and PD (Ethell et al., 2006). One study reported that the presence of α-synuclein in hematopoietic cells is linked to aberrant activation of the adaptive immune response in PD (Shameli et al., 2016), raising considerable interest among researchers. Moreover, *α*-synuclein can be presented by microglia acting as antigen-presenting cells (APCs) to stimulate T cell responses. In addition, recent evidence indicates that astrocytes with accumulated α-synuclein may also function as APCs, thereby promoting T cell activation and amplifying neuroinflammation (Rostami et al., 2020). In PD, several studies have also reported an increased proportion of circulating CD8^+^ T cells in peripheral blood. Furthermore, robust infiltration of CD8^+^ T cells has been observed in the absence of dopaminergic neuronal loss when α-synuclein pathology is not present. In contrast, during later disease stages, CD8^+^ T cell infiltration coincides with α-synuclein accumulation and subsequent neuronal degeneration (Galiano-Landeira et al., 2020). Collectively, these findings suggest that CD8^+^ T cell activation contributes to neuronal death in association with α-synuclein pathology.

## 8 Sympathetic nervous system role in modulating peripheral immune responses in PD

The sympathetic nervous system (SNS) is a major component of the autonomic nervous system and plays a pivotal role in maintaining homeostasis (Martinez-Sanchez et al., 2022). The SNS releases neurotransmitters such as norepinephrine, which bind to receptors on immune cells, influencing immune responses (Chhatar and Lal, 2021).

### 8.1 Adrenergic signaling and immune modulation

Under normal physiological conditions, stressors influence the SNS to release catecholamine from the adrenal glands (Barel et al., 2018). These neurotransmitters interact with adrenergic receptors on various immune cells, modifying their function (Chhatar and Lal, 2021). For example, catecholamines, including epinephrine and norepinephrine, bind to adrenergic receptors in the gastrointestinal tract (GIT), thereby altering gut motility, the immune response, and the microbiome composition (Leigh et al., 2023). Chronic stress and dysregulated SNS activity lead to sustained NE release, which alters immune homeostasis and promotes systemic inflammation (Sic et al., 2024; Tong et al., 2023).

Due to sustained NE release, their binding to β2-ARs significantly increases and initiates increased intracellular signaling cascades, primarily through the cyclic AMP (cAMP)–protein kinase A (PKA) pathway (Galaz-Montoya et al., 2017). In many cases, a signaling switch occurs in β2-ARs, shifting from the classical cAMP–PKA pathway to alternative pathways such as the MAPKpathway (Lorton and Bellinger, 2015). This phenomenon promotes the release of pro-inflammatory cytokines such as TNF-*α*, IL-6, and IL-1β, contributing to persistent neuroinflammation (Lorton and Bellinger, 2015). SNS activation following catecholamine binding also increases the levels of adhesion molecules and chemokine receptors, influencing neutrophil trafficking and shaping inflammatory responses (Wahle et al., 2005).

### 8.2 Integration with the hypothalamic–pituitary–adrenal (HPA) axis

The HPA axis works closely with the SNS and forms a tightly regulated neuroendocrine–immune interface (Palego et al., 2021). During stress, the hypothalamus releases corticotropin-releasing hormone (CRH), which stimulates adrenocorticotropic hormone (ACTH) secretion from the pituitary gland (Goel et al., 2025). This pathway is activated by the release of glucocorticoids (cortisol) from the adrenal gland (Goel et al., 2025; Raise-Abdullahi et al., 2023) Elevated cortisol levels may precipitate hypercortisolemia, which in turn stimulates the release of pro-inflammatory cytokines such as TNF-α (Ravi et al., 2021).

Glucocorticoids typically have anti-inflammatory effects through the inhibition of NF-κB signaling and promote anti-inflammatory gene expression (Reichardt et al., 2021). However, prolonged exposure to glucocorticoids can induce glucocorticoid resistance in immune cells (Sevilla et al., 2021). Due to this desensitization, their ability to resolve inflammation significantly decreases (Sevilla et al., 2021). This further contributes to sustained immune activation. Therefore, the dysregulation of SNS–HPA axis interplay is considered a key player in maintaining a persistent state of inflammation (Palego et al., 2021).

Finally, epidemiological studies have indicated that chronic psychological stress is a modifiable risk factor for PD (Berk et al., 2023) possibly through its impact on SNS-mediated immune dysregulation. Neuroprotective benefits can be promoted if peripheral immune responses in the SNS are modified. Beta-adrenergic antagonists such as propranolol have been well explored for their anti-inflammatory potential, which is beneficial in the context of PD (Berk et al., 2023; Inchiosa, 2024). Additionally, stress-reduction interventions, including mindfulness-based stress reduction, cognitive behavioral therapy, and physical exercise, may decrease sympathetic drive.

## 9 Clinical translation to biomarkers: current findings and challenges

From clinical and experimental data, it has been found that the pathogenesis processes leading to PD begin in the human body more than several years before the onset of motor symptoms and clinical diagnosis, at which point we can normally show signs and symptoms. It has been suggested that reliable biomarkers for early detection are also very challenging as there is no effective treatment for early detection. However, it is widely accepted that α-synuclein aggregates represent the most significant component of PD, with the resultant condition being characterized by neuroinflammation or neuronal dysfunction. The involvement of α-syn aggregation may activate the microglia and subsequently activation of receptor systems such as the Toll-like receptor (TLR) system. According to the study, blocking or depletion of TLR2 has been shown to decrease or attenuate the levels of cytokines released by microglial cell *in vitro* of PD overexpressing α-syn (Kim et al., 2013). Similar data obtained when TLR4 knockout mice treated with recombinant α-syn (Fellner et al., 2013). From this evidence it has been suggested targeting α-syn can be used as a biomarker to identify PD.

Furthermore, research conducted within the CNS has indicated that CR3/43 and EBM11 can function as biomarkers, exhibiting elevated levels in the regions of neuronal and neurite damage (Banati et al., 1998). A recent positron emission tomography (PET) study found that CSF markers of microglial activation (sTREM2, YKL-40) were associated with motor severity and TSPO-PET binding in the striatum, which is probably a potential biomarker for PD (Al-Abdulrasul et al., 2025).

However, translating these neuroimmune insights into clinical tools poses both promise and challenges. The limitations of the study are as follows: the blood–brain barrier is not fully penetrated, the cohort is heterogeneous, the prevalence of some immune signatures is low, there is a lack of assay standardization, and the temporal window during which immune modulation would be disease-modifying is uncertain.

## 10 Summary and future directions

Elucidating the involvement of the immune system in PD is crucial for elucidating its pathogenesis and discovering new therapeutic strategies. This study reinforces the idea that interactions between peripheral and central immune mechanisms play a role in PD progression. It is distinguished by its comprehensive focus on both adaptive and innate elements of the peripheral immune system.

Further investigations are essential to elucidate the specific molecular mechanisms of PD, establish validated biomarkers, and develop targeted therapies through both animal experiments and human studies. A central challenge is the incomplete understanding of the molecular basis governing the reciprocal interaction between the peripheral and central immune systems. While numerous molecules associated with immune regulation have been identified, these biomarkers have yet to be sufficiently validated and are currently inadequate for reliable clinical application, particularly in predicting disease onset or progression. Much of the existing research remains in the early experimental stages, with limited evaluation across large, diverse cohorts to ensure reproducibility and consistency. However, advancements in multiomics technologies—encompassing genomics, transcriptomics, and proteomics—combined with artificial intelligence offer growing potential for unraveling these complex interactions and identifying reliable biomarkers.

## 11 Conclusion

Together, this review highlights the involvement of both central immune cells, including microglia and astrocytes, and peripheral immune cells, such as macrophages and T and B lymphocytes, in the pathogenesis and progression of PD. The interaction between the central and peripheral immune systems is influenced by various regulatory mechanisms, including GALT signaling, epigenetic modifications, sensory neurons and the sympathetic nervous system. Future research should prioritize the application of advanced technologies to identify reliable biomarkers and develop targeted immunotherapeutic strategies.

## Glossary

ACTH: Adrenocorticotropic hormone
AD: Alzheimer’s disease
*α*-syn: α-synuclein
AP-2: Activator Protein 2
ATP: Adenosine Triphosphate
BBB: Blood Brain Barrier
β2-Ars: Beta-2 adrenergic receptor
cAMP: Cyclic adenosine monophosphate
CCR2: C-C chemokine receptor 2
CCL2: Chemokine (C-C motif) ligand 2
CGRP: Calcitonin gene-related peptide
CNS: Central Nervous System
CpG: Cytosine-phosphate-guanine
CREB: Cyclic-AMP response element binding
CRH: Corticotropin-releasing hormone
CTLA-4: Cytotoxic T-Lymphocyte-Associated protein 4
DA: Dopaminergic
DAMP: Damage-associated molecular pattern
DNA: Deoxyribonucleic acid
ENS: Enteric nervous system
ERK: Extracellular Signal-Regulated Kinase
GALT: Gut-Associated Lymphoid Tissue
HAT: Histone acetyltransferases
HDAC: Histone deacetylases
HPA: Hypothalamic–Pituitary–Adrenal
IFN-*γ*: Interferon gamma
IL1β: Interleukin-1 beta
IL4: Interleukin-4
IL6: Interleukin-6
IL10: Interleukin-10
LB: Lewy Body
LPS: Lipopolysaccharide
LRRK2: Leucine-rich repeat kinase 2
MAPK: Mitogen-activated protein kinase
MHC: Major Histocompatibility Complex
NADPH: Nicotinamide Adenine Dinucleotide Phosphate
NE: Norepinephrine
NF-κB: Nuclear Factor kappa-light-chain-enhancer of activated B cells
NK1R: Neurokinin-1 receptor
NLRP3: NLR family pyrin domain containing 3
PAMP: Pathogen-associated molecular pattern
PARK2: Parkinson protein 2, E3 ubiquitin protein ligase (parkin)
PD: Parkinson’s Disease
PINK1: PTEN-induced kinase 1
PKA: Protein Kinase A
PP: Peyer’s patches
PP1γ: Protein phosphatase 1γ
PRRs: Pattern recognition receptors
RNA: Ribonucleic acid
ROS: Reactive Oxygen Species
SCFA: Short-chain fatty acid
SNc: Substantia nigra pars compacta
SNCA: Synuclein Alpha
SNS: Sympathetic nervous system
SP: Substance P
Sp1: Specificity protein 1
TLRs: Toll-like receptors
TNFα: Tumor Necrosis Factor alpha
TRPV1: Transient receptor potential vanilloid 1
VEGF-A: Vascular endothelial growth factor A
